# On the Possibility of Calculating Entropy, Free Energy, and Enthalpy of Vitreous Substances

**DOI:** 10.3390/e20030187

**Published:** 2018-03-11

**Authors:** Sergei V. Nemilov

**Affiliations:** Saint-Petersburg National Research University of Information Technologies, Mechanics and Optics (ITMO University), 4 Birzhevaya linia, 199034 St. Petersburg, Russia; nemilovsv@yandex.ru

**Keywords:** entropy, statistical physics, thermodynamics of the vitreous state, third law of thermodynamics, glass transition, structure of glasses

## Abstract

A critical analysis for the arguments in support of, and against, the traditional approach to thermodynamics of vitreous state is provided. In this approach one presumes that there is a continuous variation of the entropy in the glass-liquid transition temperature range, or a “*continuous entropy approach*” towards 0 K which produces a positive value of the entropy at *T* → 0 K. I find that arguments given against this traditional approach use a different understanding of the thermodynamics of glass transition on cooling a liquid, because it suggests a discontinuity or “*entropy loss approach*” in the variation of entropy in the glass-liquid transition range. That is based on: (1) an unjustifiable use of the classical Boltzmann statistics for interpreting the value of entropy at absolute zero; (2) the rejection of thermodynamic analysis of systems with broken ergodicity, even though the possibility of analogous analysis was proposed already by Gibbs; (3) the possibility of a finite change in entropy of a system without absorption or release of heat; and, (4) describing the thermodynamic properties of glasses in the framework of functions, instead of functionals. The last one is necessary because for glasses the entropy and enthalpy are not functions of the state, but functionals, as defined by Gibbs’ in his classification.

## 1. Introduction

The possibility of calculating the thermodynamic functions for substances in and out of equilibrium states has been debated for almost 100 years, especially because the entropy of some solids, e.g., crystals of CO, N_2_O, glassy state of glycerol, glucose, etc., at *T* → 0 K were much greater than zero, and that violates the Third Law of Thermodynamics. A review of the first papers discussing this subject was published by Wilks [1]. Explanations of positive values of entropy at absolute zero (residual entropy) for crystals led to the development of molecular structural models, the first given by Pauling [2]. Since the 1930s, numerous studies have been performed by calorimetry in order to determine the residual entropy of a substance, *S*_0_ in the vitreous state and providing structural models for the magnitude of *S*_0_ [3].

In all calculations of entropy of glasses, the integration of glass heat capacity above 0 K was used up to glass transition temperature *T*_g_, above which the glass becomes a metastable supercooled liquid, and thereafter up to the equilibrium freezing point of the liquid or the melting point of its crystal:

(1) $$S_{T_{m}}=S_{0}+\int_{0}^{T_{m}}\frac{C_{p}}{T}dT$$

**Figure 1:**
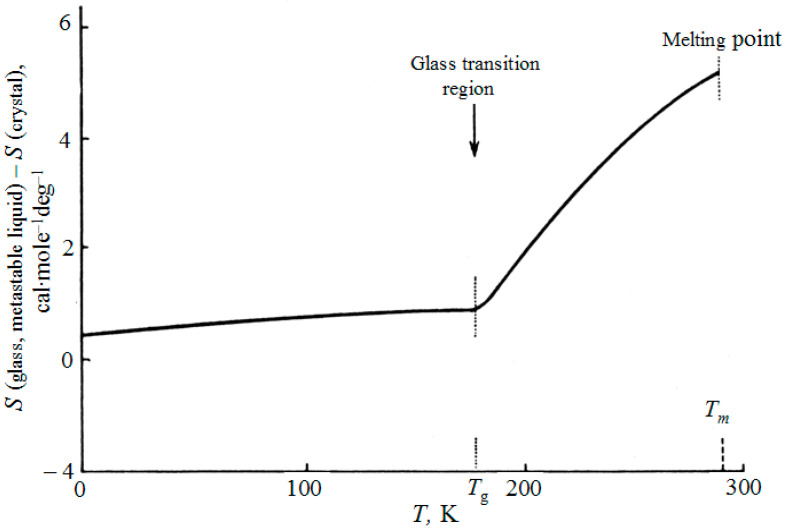
The difference between entropy of glass (below *T_g_*), metastable liquid (above *T_g_*), and the entropy of crystal (glycerol). Adapted from [1] where the experimental data were taken from the paper by F. Simon and F. Lange (1926).

The integration constant *S*_0_ is found using a similar integral, when the liquid at *T_m_* is obtained by heating the crystal, and with known entropy of melting. In this case we obtain the entropy difference between the vitreous and crystalline states (see Figure 1):

∆*S*_0_ (gl. − cr.) = *S*_0_ (glass) − *S*_0_ (crystal) (2)

This calculation supposes the *continuous variation* of entropy within the glass-liquid transition region (“continuous entropy approach”).

In 2008, a discussion on this problem was held in Trencin (Slovakia), initiated by the publication of papers by Gupta and Mauro and other authors in reputable journals. These authors insisted that the entropy of glass at *T* → 0 K tends to zero and therefore the usual thermodynamic relations that lead to *S*_0_ > 0 at *T* → 0 K are inapplicable. They insisted on the possibility of *entropy decrease* at liquid to glass transition. The final variants of the papers submitted by the authors (but not the discussion given in the form of questions and answers) have been published (see [4]), along with the point of view of Gupta and Mauro [5]. The author of the present paper took part in this discussion as the opponent of Gupta and Mauro’s view [6]. The participants of this discussion used a broad argumentation, the details of which are not worth considering here. Later this discussion continued in other places with different participants, but the details are not published. Apparently, no new evidence in favor of the known points of view has appeared. Gupta [7] and Mauro with Smedskjiaer [8] recently published two articles, which again summarized their point of view of *entropy decrease* on liquid to glass formation.

In my opinion, four most important inter-related points were missed in the discussion, for various reasons. These points are related to the justification of the eligibility of the concepts, of the terms and the models used, and their relevance to the entropy continuity or entropy decrease through the liquid-glass transition range. Their justified denial or acceptance could resolve the issue whether *S*_0_ = 0, at *T* → 0 K, or *S*_0_ > 0, at *T* → 0 K. These points are: (1) the impossibility of using classical (Boltzmann) statistics to justify the Third Law of Thermodynamics; (2) the possibility of applying thermodynamics to non-ergodic systems; (3) the impossibility of a finite change in entropy of a system without absorption or release of heat; (4) the possibility of thermodynamic calculations for the vitreous state. Before discussing their significance further, it is necessary to briefly recall some features of the vitreous state and their history and analysis.

## 2. Features of the Vitreous State

Glasses obtained by supercooling or pressurizing a liquid, or amorphous solids obtained by vapor condensation show properties that are stable over a period of time, whose duration is dictated by one’s observation time. These properties are the thermodynamic coefficients (heat capacity, compressibility, thermal expansion coefficient), and the refractive index, volume, etc. In practice, glass shows the properties of a solid only on a certain time scale.

Nernst, while formulating the Heat Theorem [9], did not distinguish this state, so for many years the majority of scientists still believed that, just like crystals, the entropy at 0 K is zero for glasses. However, later, after a reformulation of the Theorem by Planck and clarification of the validity of the theorem by taking into account the history of the glass and crystal states, it became obvious that the state of a glass cannot be a state connected by reversible transitions with the stable or metastable states. It is the *reversibility* of states that is a necessary test for the validity of the Nernst Heat Theorem for the states under consideration. However, in this case, the Third Law of Thermodynamics loses its generality and should be read as the Principle of Unattainability of absolute zero [10]. In this case, the glasses fall out of the number of states for which the entropy at *T* → 0 K would have to disappear. This principle is valid for all substances and for all states of matter (crystalline, amorphous and others). This principle is the consequence of the First and the Second Laws of thermodynamics. The best brief review of this problem was given by Haase [11].

Figure 1 illustrates the accepted *experimental* method of finding the glass transition temperature *T_g_* as the point of intersection of two lines: the line extended from the glass region and the line for the region of metastable liquid. The transition between these branches when various properties are measured in cooling (the volume, the heat content, etc.) is always blurred; inside this narrow area, one can not speak only of glass or only about a metastable liquid.

The appearance of the vitreous state (glass transition) is described theoretically in the framework of the origination of nonequilibrium state as a process of freezing-in the internal structure of matter (see, for example, [3] and Prigogine and Defay [12]). Freezing-in means the case where the change of a usual thermodynamic parameter (temperature or pressure, or both) does not cause variation of the structure of the substance which would correspond to the value of this parameter. The reason is the occurrence of a very strong retardation of the processes of structural relaxation with decrease in the temperature [3].

The relationship between the changes in the thermodynamic coefficients on transition from the nonequilibrium state (glass) to the equilibrium (say, metastable liquid) at the glass transition temperature *T_g_* (*T_g_* is inside the transition temperature range) is described by the equation that is formally similar to the Ehrenfest relation for second-order phase transitions. This similarity is not real but apparent, since in such phase transitions one stable structure is replaced by another stable structure, and the transition point is uniquely determined. In contrast, in the course of the liquid-glass transition, the rate of temperature change affects the position of the interval of glass transition within the thermodynamic temperature scale. An analogous statement is also valid for glass transition with pressure change. For this reason, the frozen-in glass structure must be different in each specific case. Glass transition is a transition neither of the first nor of the second order. All properties of the vitreous state are associated with the energy and nature of the arrangement of the particles which must be different according to the prehistory of the state. The latter circumstance is mainly accepted by both parties who participated in the above mentioned discussion held at Trencin.

All modern theories of glass transition take this into account (both the theories describing this process with the linear temperature change in time and with a periodic change). For this reason, the kinetic theories of glass transition and the dynamic theory of liquids are in complete agreement with each other (see [3,13]). In the dynamic theory of liquids, a solid-like state arises at a certain definite ratio of the frequency of the periodic external action on a system and the time of its structural relaxation. This theory shows that, when the external parameters of the whole system (temperature and pressure) are constant, the periodic action on the liquid can reveal either only the elastic properties of a liquid (instantaneous elastic moduli, instantaneous thermodynamic coefficients) or equilibrium ones (Newtonian viscosity, equilibrium thermodynamic coefficients). The validity of the dynamic theory of liquids is currently not seen as a subject for revision. These questions are considered most consistently in [3,13], and here the details are omitted.

Glass is a solid with certain properties (for example, volume). These properties practically do not change, if the accuracy of their measurements allows such conclusions to be drawn. This case is realized when the duration of observation τ_obs_ is substantially shorter than the time of the structural relaxation, τ_rel._ (τ_obs._ << τ_rel._). For this reason, it is possible to measure the temperature with glass thermometers (as used by Joule) and use lenses and reflectors made of glass in the practice of astronomical observations. The definiteness of the properties of glass as the material enabled the successful use of glasses possible for scientific and technical purposes for several centuries of human history, including thermodynamic studies (see, for example, Joule’s papers [14]).

By changing the cooling rate within the temperature range near the glass transition temperature *T_g_*, it is possible to vary the *T_g_* value itself, and a property of the obtained glass, and thus to control this property (this is a usual process of high-temperature annealing in glass manufacturing). In these processes, the characteristic time of structural relaxation can be as long as several days or months. The spectrum of relaxation processes is very wide. Most often, relaxation of properties is not described by a simple exponential function, which, apparently, is associated with a set of relaxation times and with a sequence of relaxation paths. Probably, this complexity is the reason of stretched-exponent dependence for relaxation of properties (Kohlrausch relaxation law). Depending on the glass transition temperature, the chemical equilibria between the components of a system can be quenched at different levels, which predetermine the different chemical composition of the various regions of a substance.

The variability of the scales of glass thermometers and the refractive indexes of optical details over time intervals estimated in *decades* (at ordinary temperature) undoubtedly always characterizes the solid glass as a very unstable state [15]. These processes would have to be determined by the characteristic times of structural relaxation of the order of 10^7^ years, if the developments of structural change processes are of the same type as in the *T*_g_ region. Practically, here the relaxation times, however, are of the order of a year, several years or tens of years. This is the evidence for the fact that the vitreous state should be classified as an *absolutely unstable (labile) state of matter* [3].

In a metastable liquid above the glass transition temperature, changes in the structure of the system immediately follow changes in temperature; the probability of such changes is very large. Below the glass transition temperature the probability of structural changes becomes extremely small. Is it possible, therefore, for the vitreous state to define certain values of entropy, heat content and free energy, which could characterize the vitreous state as a thermodynamically determined state of matter?

With this background to glass and glass formation, it is necessary to formulate the essence of theoretical “*continuous entropy approach*” to glass transition problem. As mentioned in Section 1: Introduction, this approach corresponds to the principle of entropy calculation by means of equations like (1) and (2).

## 3. Main Features of Glass Transition in the Framework of “Continuous Entropy Approach”

**Figure 2:**
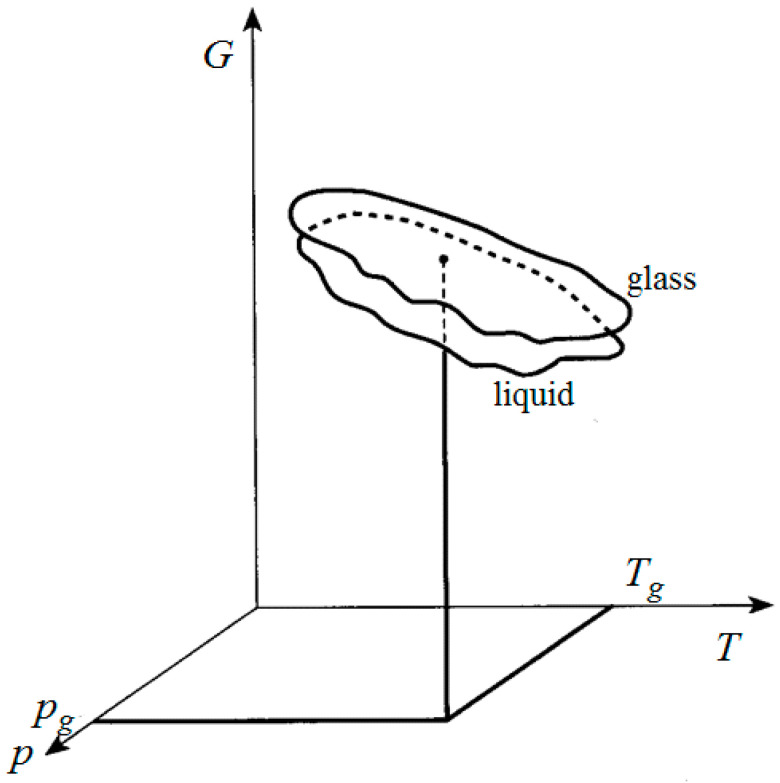
The touching of the free energy surfaces of liquid and glass at the point *T_g_* (from Nemilov [3]).

Experimentally, the change in the state of a liquid upon cooling and its transition to a state with the frozen-in structure (glass) is not accompanied by exothermic or endothermic effects. This requires the use of kinetic modeling that is not complicated by discrete-type structural processes associated with the same discrete entropy changes. This feature of glass transition is completely taken into account in the kinetic theory of this event (see [3]). Formation of the vitreous state is inevitably associated with the appearance of excess free energy. The excess free energy (and entropy) of the frozen state appears solely due to the difference in the thermodynamic coefficients (compressibility, heat capacity, and thermal expansion coefficient) of the initial and generated states. The appearance of free energy difference of these states has been explained after expanding this difference in a Taylor series at *continuous* variation of entropy solely by the difference of second derivatives of free energy of both states at *T_g_* (for a mathematical description, see [3]). These coefficients are of a structural nature; their values can vary with temperature according to kinetic reasons, as the structure itself varies, which leads to glass transition. Therefore, the surfaces of free energy of the initial liquid and glass in the kinetic theory only *touch* each other at the glass transition point, but do not intersect (Figure 2). The glass transition here is a continuous process; the entropy changes continuously. The main feature of this approach is that *at the glass transition point* it is meaningless to talk about the simultaneous existence of a vitreous state and the state of a metastable liquid. The vitreous state appears only *outside* the point indicated as *T*_g_, within its neighborhood. An excess of free energy arises only outside this point, and the process of transition to an equilibrium state develops as a natural one. The driving force of this process is the excess free energy [3,13].

## 4. Impossibility of Using Classical Statistics to Justify the Zero Value of the Entropy of Glass at *T* → 0 K

The authors of [5,8] and their colleagues have applied the classical Boltzmann statistics for finding the entropy of a non-isolated non-equilibrium system in condensed state. It is known that classical statistics as an approximation is valid for the states at high temperatures with low density (e.g., for gases). Gupta, Mauro and their colleagues use the fact of freezing liquid system at glass transition in a unique and non-equilibrium structural state to conclude that at absolute zero the entropy must be zero, since the macroscopic state is unique: “in our view, at *T* = 0, a system is kinetically trapped in a single configurational state and cannot explore other degenerate states …” [5]. This result, however, is predetermined by the method of *classical* statistics which they used. The possibility of nonzero entropy value at *T* → 0 K was presumed from the beginning starting from the premise that all particles are indistinguishable. The fallacy of such use of classical statistics was mentioned by almost everyone who had compared the possibilities of classical and quantum statistics to explain their applicability to the understanding of the Nernst Heat Theorem. For example, Landau and Lifshitz [16] wrote: “It should be emphasized that this theorem is a deduction from quantum statistics, in which the concept of discrete quantum states is of essential importance. The theorem cannot be proved in purely classical statistics, where the entropy is determined only to within an arbitrary additive constant”.

It was Gibbs, who suggested, in the statistical description of the system consisted of a large number of particles, that there might exist *groups of particles that differ from the others* by the number of degrees of freedom. Making the allowance for such groups and the number of permutations within them alters the classical expression for the volume of the phase space of the system ([17], Vol. 2, *Elementary Principles in Statistical Mechanics,* Introduction; Chapter VIII). Thus, the *difference between particles* may be introduced in classical statistics and this statistics partially loses the original meaning.

In Bose-Einstein or Fermi-Dirac quantum statistics, the different groups of particles are those that differ in quantum number or spin. The phase volume is equal to 1 for *T* → 0 K only if the particles are indistinguishable (in Bose-Einstein statistics with respect to the zeroth energy of vibrations). However, this is possible only if each microvolume of matter has the same structure, composition, and energy. Only in this case, with a decrease in temperature, degeneracy progresses and the entropy of the macrostate can tend to become zero. In glasses, as mentioned above, a complete equivalence of all microregions of a body is impossible. *Therefore, application of classical statistics to glasses is not valid*. It is known that both the Bose-Einstein and Fermi-Dirac quantum statistics approach the formalism of classical statistics only at high temperatures and low densities. An excellent analysis of the differences between classical and the both quantum statistics is given in [10,18].

It is interesting to note that among the systems in which the centers of gravity of molecules have a regular (crystal-like) disposition, the class of disordered systems (glassy crystals) exists, which differs from usual crystal structures by the disorder of the mutual orientation of the molecules. Obviously, orientational ordering can be attributed to a feature that distinguishes the state of each molecule from that of the other molecules. This parameter is frozen as it is frozen in the vitreous state. In these systems, the glass-like transition manifests itself in the same way as the glass-liquid transition. This leads to the conservation of the difference between the particles as *T* → 0 K. A consequence is the existence of nonzero entropy as *T* → 0 K [3,19]. This case resembles the models proposed by Pauling [2].

## 5. Possibility of Applying Thermodynamics to Nonergodic Systems

The authors of [5,8] and their colleagues, who adhere to the same point of view, deny the applicability of Boltzmann statistics for determining the entropy of a nonequilibrium system. They believe that the concept of Boltzmann entropy is applicable only to equilibrium systems for which the ergodic theorem is valid. This theorem presupposes that all microstates are realizable for a sufficiently long period of time.

We were convinced that the state of a liquid, depending on the ratio of the duration of observation and the time of structural relaxation, could correspond to different values of the properties. Apparently, this point of view is shared by many participants of the discussion [4]. These properties of glasses can be practically unchanged (this is obvious at low temperatures), which indicates the conservation of the average coordinates (the arrangement of atoms) and the dynamics of their motions. During a *very long* observation time, the coordinates (position) and momenta of particles will vary measurably in such a system. The system may be completely ergodic and will vary following the state parameters (this is a liquid above the glass transition temperature).

Both states (fixed particle coordinates with free realization of all admissible momenta, and free realization of all admissible coordinates and associated momenta) can be observed *separately* if the *probabilities* of their observation differ substantially according to the observation conditions (see also [17], Chapter VI).

Ergodic theorem was developed after the Boltzmann published his work in the 1870s essentially as a purely mathematical theory in the framework of the general theory of *dynamical systems*. Actually, Gibbs applied a statistical consideration to both *equilibrium* and *non-equilibrium* systems ([17]; Vol. 2, *Elementary Principles in Statistical Mechanics*: Chapter XI, Theorem III; Chapter XII). He considered it possible to compare both *equilibrium* and *non-equilibrium* states, however, he did not use the term “ergodicity”. The system is *non-equilibrium*, if the canonical distribution of points in the phase space is not realized. In this case the free energy of the system is *higher* than the energy of the system with the canonical distribution.

Gibbs considered the possibility of a *separate* description of the phase volume constituents: the configurational volume connected with the coordinates (*q*), and the volume in the space of velocities (momenta, *p*), which he associated with the corresponding *entropy* components: *S_q_* + *S_p_* = *S* ([17]; Vol. 2, Elementary Principles in Statistical Mechanics: Chapter VI). Gibbs suggested the mentioned phase volumes as a *dynamic* measure of the *volume of configurations* and a *dynamic* measure of the *volume of the velocity space*. However, Gibbs believed that the volume in the velocity space has to correspond to the definite volume in the configurational space (i.e., the momenta must be related to the definite coordinates). Correspondingly, according to Gibbs, the entropy of a “canonical system” appears to be larger than the entropy of the system without canonical distribution. Gibbs did only the *comparison* of these cases. However, the states under this consideration cannot be realized reversibly one into the other, it was only an conceptual comparison!

Thus, *Gibbs himself did not demand the existence of ergodicity for a system in order to have some entropy value* [17] (as was mentioned, he did not use this term as many others now do). The notions of “canonical ensemble” and “ergodic” system, strictly speaking, are not identical. The only thing required when considering the entropy of a system is the constancy of the phase volume (see too [20]). For glass, especially at low temperatures, this is certainly true within the framework in which allows realization of the probable statistical distribution of coordinates and momenta.

In our time, the questions of ergodicity were discussed by many physicists, including Academician Bogolyubov in Russia. He came to the conclusion that to justify the relations of statistical thermodynamics, ergodicity is not necessary, and the shortcomings of this principle are clearly manifested in the study of dynamical systems of quantum mechanics [20]. Bogolyubov wrote: “Let me remark that the presence of property of kneading at any finite *N*, *V* for the foundation of statistical thermodynamics is absolutely dispensable. Important is only the corresponding behavior of finite values of macroscopic quantities at t → ∞ after realization of limiting transition of statistical mechanics, namely, *N* → ∞, *V* → ∞, *N*/*V* = $\bar{n}$ = const. The insufficiency of ergodic theory in its modern formulation becomes especially clear at the consideration of dynamic systems of quantum mechanics”. Thus, the principle of ergodicity used in thermodynamic discussion of the existence of certain values of entropy is not necessary. Otherwise the assertions of [2,8] would require proof.

## 6. Impossibility of a Finite Entropy Change of a System at *T_g_* in the “Entropy Loss Approach”

The possibility of calculating entropy in thermodynamics (if we forget for a moment the necessity to obey the principles of statistical physics) is defined as the adherence of the reversibility of absorption and release of heat by the system. When studying the vitreous state in its genesis, the solution of this problem is simple. Reversibility is always realizable if the heat capacity of a *glass* is measured *very quickly* (so that structural relaxation does not occur) from arbitrarily low, experimentally available temperatures up to *T_g_*. The measurements are also realizable in metastable *liquid*, if they are taken *slowly* enough for completing the relaxation processes. Thus, the entire uncertainty in the magnitude of the entropy change upon glass transition reduces to the vanishing temperature interval δ*T_g_* in the vicinity of *T_g_*. Within this interval, which can be made arbitrarily small, there is no reversibility of the states, and this point is ruled out from the calculations [6]. Since this calculation procedure is not related to actual physical processes, it is only an imaginary operation! In this way, to calculate the entropy of the liquid at the equilibrium temperature of melting, *T_m_*, the integration constant is introduced. In doing so, one does not require knowledge of the entropy of a real glass at *T* → 0 K. It always gives the difference in the entropies of this state (glass), and of the state under comparison (here it is a crystal) at *T* → 0 K. The liquid at *T_m_* has entropy, enthalpy and free energy, which really are the *functions of state* and do not depend on the thermal path by which the liquid was obtained. The liquid at *T_m_* can be prepared either by heating a crystal from 0 K with the subsequent melting, or by heating the glass of the same composition from 0 K.

The main feature of the approach of the opponents of traditional approach to the thermodynamics of the vitreous state, and supporters of the “entropy loss approach” is that at the glass transition point *T_g_* is possible to have the existence of both the glass and the metastable liquid with the different entropies: «glass and liquid are different macrostates at *T*_g_» [5]. As mentioned above, in the classical approach this is nonsense, both states cannot exist one near the other at the same temperature. So, the process of glass formation in the works of the authors [5] is considered as the result of an artificial (“non-spontaneous”) action on a system that develops in a time period shorter than the structural relaxation time. “Liquid-glass transition is an ergodicity-breaking process caused by an external intervention in the form of finite observation time” [5]. As a result, at *T*_g_, the system transforms to a state with *broken ergodicity*, which, in the opinion of Gupta and Mauro, contradicts the possibility of equivalence of the entropies of glass and liquid, and the inequality is necessary *S_T_*_g_ (glass) < *S_T_*_g_ (liquid).

So, the opponents of nonzero entropy of glass at *T* → 0 K agree that, at *T_g_*, the volume and enthalpy of the glass change continuously (∆*V_T_*_g_ = 0, ∆*H_T_*_g_ = 0) [5], but the entropies are different, exclusively, as the result of the short external action; this statement raises objections. They believe that, if the state of glass is realized under conditions of violation of the reversibility of states in a time shorter than the time of structural relaxation, then the difference in the entropy of the glass and the glass-forming liquid at *T_g_* (which is considered as a point) may correspond to the *disappearance* of entropy (*S*_glass_(*T_g_*) < *S*_liquid_(*T_g_*)) [5]. This inequality served later as the starting point for further proof the inequality *S*_glass_(*T →* 0) = 0.

The objection is that if the volume of a closed system (not isolated), is kept constant at *T_g_*, the change in entropy at *T_g_* without release of heat or its absorption is impossible, according the Second Law.

This entropy loss view [5] was more particularly criticized by Goldstein [21,22,23], who concluded that the theory of authors [5] leads to the possibility of the existence of *perpetuum mobile* of the 2nd kind. The authors [5] believe, however, that in his arguments, Goldstein mistakenly took a non-zero (residual) entropy of the glass for a really existing entropy. “*In our view, at T = 0, a system is kinetically trapped in a single configurational state and cannot explore other degenerate states, and that any transition has to freeze out at a sufficiently low temperature, … the extrapolation of the calorimetric entropy to absolute zero* … *gives the ergodic entropy and does not represent the real entropy of the system at 0 K. Therefore, for kinetic reasons, the real (non-ergodic) entropy of a glass is zero at absolute zero*” [5]*.* In this objection, only old arguments were repeated obtrusively, without any new foundation.

## 7. Possibility of Thermodynamic Calculations for the Vitreous State

When someone uses the terms “Gibbs free energy” and the relationships connecting this energy with enthalpy and entropy, it must not be forgotten that these functions for glass are not *the functions of state* ([17]; Vol. 1, *Thermodynamics: Graphical methods in the thermodynamics of fluids*). Mauro and Smedskjiaer draw a sharp distinction between the generality of physical approximations in the description of equilibrium and nonequilibrium states [8]. They did not agree that “*… introduction of one or more additional parameters, called “order parameters” responsible for describing the nonequilibruum state of glass*” is, in fact, a scientific approximation. They believe that it does not correspond to the fundamental character of the physical description: “*… irreversible thermodynamics is phenomenological by design and hence not rooted in any fundamental physics*” [8]. Of course, one can agree that a complete description within the framework of statistical mechanics is preferable to other approximations. However, everything depends on the theory formulation and on the physical meaning attributed to the “additional parameter”. In the thermodynamics of the vitreous state, this is the structural parameter ξ, which, along with temperature and pressure, determines each state of the system, its entropy, energy and enthalpy: *S*(glass) = *S*(*T*,*p*,ξ), etc. In the kinetic theory of glass transition, based on this parameter (single ξ or several ξ*_i_*), the *way* of obtaining the state of a glass and changing the system properties accompanying this process is described. The order parameter is functionally equivalent to the composition parameter, which determines the state of the system in Gibbs thermodynamics. Therefore, when considering the glass transition conditions (the variational problem) and the Prigogine-Defay Ratio, one uses the theory of determinants posed on the second derivatives of Gibbs’ free energy (similar to the way it is done in Gibbs’ theory for describing phase equilibria). I was not the first to apply this approach to glass transition; in the book [3] a detailed bibliography is given. Probably, the order parameter ξ*_i_* may be connected with the topological dimension of the space containing the structure elements (see, e.g., [24]).

Gibbs wrote that, the state, if it depends on the way it is obtained, is no longer characterized by state functions as they are understood by equilibrium thermodynamics ([17]; Vol. 1, *Thermodynamics:* *Graphical methods in the thermodynamics of fluids*). However, this representation of the role of order parameters corresponds to the definition of functionals in mathematics [25]. Therefore, according to a mathematical feature, the entropy, energy and enthalpy of glasses remembering their difference from the Gibbs’ state functions, are called the functionals of state [3]. The theory of functionals has a fundamental character of physical description and is the basis of many modern branches of physics [25].

De Donder and Rysselberghe [26] and then Prigogine and Defay [12], Leontovich and Mandelstam, and others (see [3,13]) considered the internal parameter ξ (ξ*_i_*) to be *equal in rights* to the parameters of the state *T* and *p* at the description of glass transition. A system of equations, connecting the properties of a body by means of the second derivatives of energy with respect to pressure, temperature, and the parameter ξ, represents ξ as a *peer-to-peer* parameter of the theory; this parameter being as equitable as *T* and *p* in the Gibbs theory of equilibrium systems. Thus, not only variational problems are described (the appearance of excess energy), that is, the output of the system from the energy minimum during the glass transition [3], but also kinetic ones (the phenomenon of glass transition itself and relaxation to equilibrium state [13]).

The usual thermodynamic relationships determined by the energy derivatives (according to Gibbs), but supplemented by taking into account the existence of the structural (internal) parameter ξ, are also considered valid in this case, as in ordinary thermodynamics, since “*these “functions” are equivalent to functions of the elementary theory of functions of many variables*” (See “The implicit function theorem” in [25]). It is on this basis that the entropy of glasses (in comparison with the crystals) is calculated at *T* → 0. A knowledge of the latter quantity (∆*S*_0_ (gl. − cr.)) is extremely important for the calculations of thermodynamic potentials at each temperature, since the integration is required, implying the possibility of the existence of integration constant). Such calculations [3,6] made it possible to find the numerical values of the difference in entropy, free energy and enthalpy of glass and crystal at 0 K and for all temperatures in the range from 0 up to melting temperatures for various substances. The values of the entropy and excess free energy of a glass with respect to crystal were always *positive* below *T*_g_ due to the fact of the positive integration constant ∆*S*_0_ (gl. − cr.). This method of calculating entropy, free energy and enthalpy for glasses was adopted in the world practice; the results of such calculations are given in scientific articles and in all reference books on thermodynamics.

The analysis of existence of *plurality* independent *order parameters* ξ*_i_*, which determine the state of a glass, was carried on the basis of Prigogine-Defay Ratio and the Cauchy-Schwartz-Bunyakovsky theorem based on the theory of functionals (see [3]). This way avoids the erroneous non-thermodynamic “phenomenological approach”; see the criticism of this approach in [6,13]. 

Gupta [7], while criticizing the approach practiced in the glass’ thermodynamics, tried to reproduce it. He demonstrated that the free energy of a liquid below *T*_g_ is higher than the free energy of a glass produced from it. However, the reason for the meaningless of this result, which he also noted, is that this author, assuming the entropy of the glass to be zero at 0 K, did not introduce the integration constant and used model representations of another theory not included in the theory of his opponents. Thus, Gupta showed that the concept chosen by himself and his colleagues leads to a meaningless result. Very strange is an unproven assertion [7], that at thermodynamic temperature *T below* the fictive temperature *Т*_f_ (close to *T*_g_), the relaxation rate is *higher* than above it (i.e., when *T* > *Т*_f_). It should be the opposite, as *Т*_f_ determines only the height of the potential barrier of the activation process. When the melt is cooled, the height of the barrier is frozen, since the structure does not change.

An important consequence of my approach to the entropy of glass is that the main consequences of Gibbs thermodynamics allow us to obtain important results that go beyond the problem of the vitreous state. *Changes in the Gibbs free energy, as always, determine the value of the most useful work, regardless of whether this energy is a function or a functional.* This is extremely useful. Following the principle of minimizing the free energy of a disordered system, the dynamic properties (e.g., the frequency spectrum of particles motions) adapt to the genetically arising internal structure, which we can vary (see the bibliography in [3,6]). Perhaps this is the manifestation of self-organization (synergetics) in disordered systems with strong interactions.

Just as the emergence of statistical mechanics required knowledge of the molecular structure of gases, a complete statistical theory of glasses requires the knowledge of their structure at the molecular level. Numerous works were devoted to the studies of the structure of glasses in the last century, and studies of the structure continue today. If the attempts to solve this problem made throughout the entire 20th century are carefully traced, it can be seen that the glass structure cannot yet be formulated in the most general, simple and sufficiently accurate ways. 

Hopes for a full statistical description of the thermodynamic functions expressed by Mauro and Smedskjiaer [8] can be shared, but such hopes are illusory. The wide used method of computer simulation is excellent, but it always requires the use of information found empirically. Such a way cannot be considered as a pure theoretical one.

## 8. Conclusions

The main reason of the discussion between supporters and opponents of non-zero values of the entropy of glass at *T* → 0 contains in the different understanding the thermodynamic model of glass appearance from the liquid. In fact, both approaches select this model basing on different considerations. «Continuous entropy approach» from the very beginning considerers the “liquid-glass” transition corresponding to direct experimental facts consisting in monotonous change in volume and heat content during such a transition. The “entropy loss” approach ignores this experimental fact, but it exploits the concept of broken ergodicity. In the opinion of the supporters of this theory, the violation of ergodicity makes the application of the existing thermodynamic relations impossible.

The other reasons of debate are as follows: *a different choice of the theoretical basis for consideration* of the subject of the discussion, or an *axiomatic* statistical approach, *different understanding of the terms* used and their *applicability* to substances in the vitreous state. Each of the discussants chose to build the ranking of the justification of their statements.

In my opinion, the *basis* for the rejection of the existing thermodynamic approach in the works of Mauro, Gupta and their co-authors are: (1) unreasonable use of the classical Boltzmann statistics to calculate the value of entropy at absolute zero; (2) the neglect of thermodynamic analysis of systems with broken ergodicity, although the possibility of such analysis was shown by Gibbs; (3) the possibility of a finite change in entropy of closed system at constant volume without absorption or release of heat; and (4) the negation of the possibility of describing the thermodynamic functions of glasses in the framework of functional analysis. The latter’s need for glasses is due to the fact that these functions are not functions of the state, but functionals, which corresponds to Gibbs’ classification of states.

In this paper, for the first time, the principles for calculating the thermodynamic functions of glasses, which follow from the works of Josiah Willard Gibbs, are clearly formulated. If we rely upon the classical works of Gibbs, Planck, Einstein, Fermi, Prigogine, and other authors of modern physics, it is impossible to accept the limitations of the thermodynamic consideration of the vitreous state proposed by Gupta, Mauro and co-authors. Now everyone, who is interested in this problem, can make one’s own choice in accordance with one’s understanding.

Apparently, time will pass and everything will fall into place. I can fully agree with the words published by Mauro and Smedskjiaer: “*the use of the statistical approach does not affect the thermodynamic properties of the system*” [8].

In this paper I strived to avoid citing the secondary sources; the fundamental ideas by Gibbs were developed later by the excellent scientists in many countries.
